# Supplementary material for: Broadband high-Q multimode silicon concentric racetrack resonators for widely tunable Raman lasers
Source: Nat Commun. 2022 Jun 20;13:3534. doi: 10.1038/s41467-022-31244-0 (PMC9209424; doi:10.1038/s41467-022-31244-0)
Supplement: Supplementary file 1 — Supplementary Information [file 41467_2022_31244_MOESM1_ESM.pdf]

Supplementary Information

Broadband high-Q multimode silicon concentric racetrack resonators for widely tunable Raman lasers

Yaojing Zhang[†,*], Keyi Zhong[†], Xuetong Zhou, and Hon Ki Tsang[*]

Department of Electronic Engineering, The Chinese University of Hong Kong, Shatin, New Territories, Hong Kong

[†]*These authors contributed equally to this work*

**Corresponding author: yaojingzhang@cuhk.edu.hk (Y.Z.), hktsang@ee.cuhk.edu.hk (H.K.T.)*

S1. Mode coupling in a multimode single resonator.......... 2

S2. Mode coupling in a multimode concentric resonator 5

S3. Broadband high-Q multimode concentric resonator.......... 7

S4. Modes in the multimode concentric resonator.......... 10

S5. Raman lasing characteristics.......... 11

S6. Raman lasing experimental setup 13

S7. Resonator characteristics 13

S8. Comparison of silicon Raman lasers 15

S9. Photocurrent characteristics.......... 15

S10. Development of low-loss silicon nano waveguide 16

S1. Mode coupling in a multimode single resonator

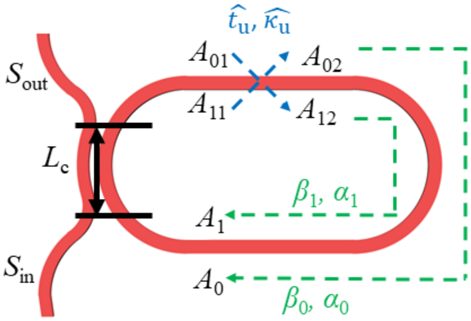

Fig. S1 | Schematic diagram of a multimode single racetrack resonator. The input pump is injected into the bus waveguide and couples to the racetrack. The mode coupling between the TE0 mode and higher-order mode happens inside the racetrack.

The loaded quality (Q) factor is a parameter that depends on the total loss of a resonant cavity, including coupling loss and propagation loss. A cavity with broadband high loaded Q factors is desired for nonlinear applications, potentially useful for enabling lower input powers by enabling high enhancement over a broadband range. Firstly, we would like to explain why mode coupling can be a problem on the loaded Q factors in a multimode single resonator.

$$\frac{\partial A_0}{\partial t} = (j\omega_0 - \frac{1}{\tau_0})A_0 - j\kappa_0 S_{in} - ju_t A_1 \quad \text{(S1)}$$

$$\frac{\partial A_1}{\partial t} = (j\omega_1 - \frac{1}{\tau_1})A_1 - j\kappa_1 S_{in} - ju_t A_0 \quad \text{(S2)}$$

For a multimode single resonator, the influence of mode coupling on loaded Q factors can be explained from equations (S1-S2) by modeling the multimode single resonator with only fundamental (TE0) mode and higher-order mode[1]. Parameters A_0 and A_1, ω_0 and ω_1, τ_0 and τ_1, κ_0 and κ_1 are the amplitudes, resonant frequencies, photon lifetimes, and coupling coefficients of the TE0 mode and higher-order mode, respectively. u_t is the mutual coupling coefficient between the two modes. S_{in} and S_{out} are the amplitudes of the input and output pump waves.

Table S1 | Parameters of the multimode single resonator.

mode	Resonant wavelength (nm)	Propagation loss α (dB/cm)	Coupling ratio $\|\kappa\|^2$ (s^{-1})	Coupling condition
TE0	1555.5	0.26	6.9×10^8	under coupled
higher-order mode	1555.54	2.2	3.6×10^7	under coupled

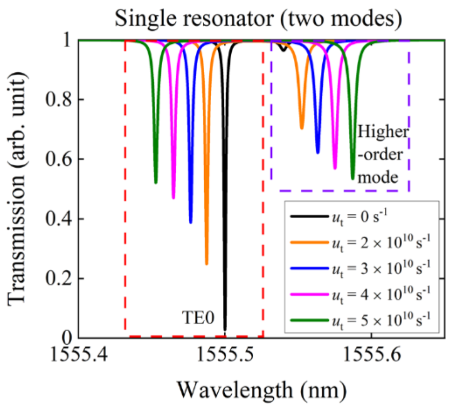

Fig. S2 | Simulated transmission spectra of the multimode single resonator with different mutual coupling coefficients u_t. The TE0 mode and higher-order mode couple to each other with different mutual coupling coefficients.

In a multimode single resonator like Fig. S1, by assuming the TE0 mode and higher-order mode with parameters listed in Table S1, we solved equations (S1-S2) with different mutual coupling coefficients and obtained the transmission spectra in Fig. S2. Here, two adjacent wavelengths instead of equal resonant wavelengths are selected to display the independent loaded Q factors of the two modes by setting the mutual coupling coefficient u_t to 0. In reality, u_t is a fixed value for a given structure. When the two resonant frequencies approach each other, amplitudes of the two modes would increase, leading to the enhancement of the coupling term $-ju_tA_0$ or $-ju_tA_1$.

Table S2 | Calculated loaded Q factors of the multimode single resonator for different mutual coupling coefficients u_t corresponding to Fig. S2.

u_t (s^{-1})	Loaded Q factor (TE0)	Loaded Q factor (higher-order mode)
0	1.0×10^6	2.9×10^5
2×10^{10}	0.69×10^6	3.4×10^5
3×10^{10}	0.61×10^6	3.6×10^5
4×10^{10}	0.57×10^6	3.8×10^5
5×10^{10}	0.54×10^6	3.9×10^5

In the simulation, we fixed the two resonant frequencies and gradually increased u_t. With the obtained transmission spectra in Fig. S2, we calculated the loaded Q factors for different u_t and summarized them in Table S2. With the increase of u_t, the loaded Q factor for TE0 mode gradually reduces. Because the resonances of the TE0 mode and a higher-order mode get closer in the wavelength domain, the TE0 mode thus suffers from additional effective loss from the coupling to the higher-order mode and thus its loaded

Q factor is reduced. The loaded Q factor of the TE0 mode can reduce by about 50% compared with that without the mode coupling as summarized in Table S2.

$$S_{\mathrm{out}} = \hat{t}_{\mathrm{in}} S_{in} - j\hat{\kappa}_0 A_0 - j\hat{\kappa}_1 A_1,\ z \in [0, L_{\mathrm{c}}] \tag{S3}$$

$$A_{01} = \hat{t}_0 A_0 - j\hat{\kappa}_0 S_{\mathrm{in}},\ z \in [0, L_{\mathrm{c}}] \tag{S4}$$

$$A_{11} = \hat{t}_1 A_1 - j\hat{\kappa}_1 S_{\mathrm{in}},\ z \in [0, L_{\mathrm{c}}] \tag{S5}$$

$$A_{02} = \hat{t}_u A_{01} - j\hat{\kappa}_{\mathrm{u}} A_{11} \tag{S6}$$

$$A_{12} = \hat{t}_{\mathrm{u}} A_{11} - j\hat{\kappa}_{\mathrm{u}} A_{01} \tag{S7}$$

$$A_0 = A_{02} \mathrm{e}^{-(j\beta_0 + \frac{\alpha_0}{2})(L - L_{\mathrm{c}})},\ z \in [L_{\mathrm{c}}, L] \tag{S8}$$

$$A_1 = A_{12} \mathrm{e}^{-(j\beta_1 + \frac{\alpha_1}{2})(L - L_{\mathrm{c}})},\ z \in [L_{\mathrm{c}}, L] \tag{S9}$$

The performances of the two modes in the multimode single resonator can also be described by equations (S3-S9) under the assumption that the mode coupling [equations (S6-S7)] occurs immediately after the interaction between the two modes with bus waveguide [equations (S3-S5)][2]. Then, the two modes propagate along the resonator with phase change and loss [equations (S8-S9)]. $\widehat{t_0}/\widehat{\kappa_0}$, $\widehat{t_1}/\widehat{\kappa_1}$, $\widehat{t_{\mathrm{u}}}/\widehat{\kappa_{\mathrm{u}}}$ are amplitudes of transmission/coupling coefficients between the TE0 mode and bus waveguide, the higher-order mode and bus waveguide, and the TE0 mode and higher-order mode, respectively. $\widehat{t_{\mathrm{in}}}$ is the amplitude of the transmission coefficient of the bus waveguide and is set to be $\sqrt{1 - \widehat{\kappa_0}^2 - \widehat{\kappa_1}^2}$. α and β are the propagation loss and constant. L_{c} is the coupling length between the bus waveguide and resonator, while L is the roundtrip length of the resonator.

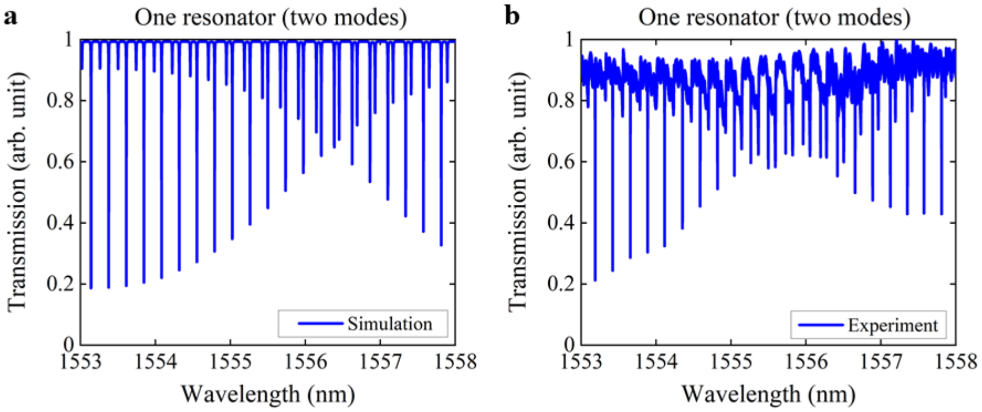

Fig. S3 | Transmission spectra of the multimode single resonator with two modes of the TE0 mode and the higher-order mode. a, From the simulation. **b,** From the experiment.

By solving equations (S3-S9), we obtained the transmission shown in Fig. S3a. We can see that the mode coupling occurs at about 1556 nm. The loaded Q factor was calculated to be 4.9 × 10^5 compared to the loaded Q factor of 1.0 × 10^6 at 1553 nm which is away from the mode coupling. There is a 51% reduction in the loaded Q factor at the mode coupling region. The experimental transmission spectrum in Fig. S3b agreed well with the theoretical prediction in Fig. S3a.

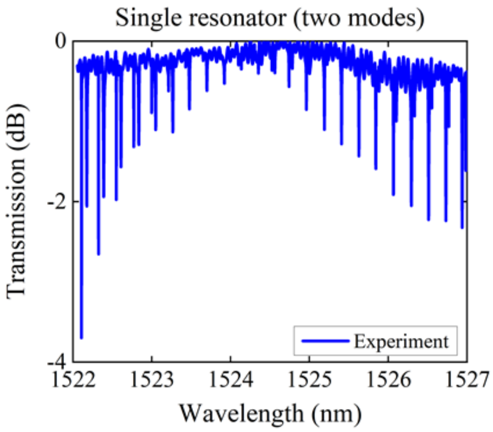

Fig. S4 | Experimental transmission spectrum from 1522 nm to 1527 nm of the multimode single resonator with two modes. The TE0 mode resonances are largely coupled with the higher-order modes.

Some highly affected TE0 mode resonances by the mode coupling can occur as the example in Fig. S4, the loaded Q factor decreased to 2.1 × 10^5 at a wavelength of 1524.1 nm compared to that of 1.1 × 10^6 in the non-mode coupling region. There is an 81% reduction of the loaded Q factor and 10 times larger loss at this resonance.

S2. Mode coupling in a multimode concentric resonator

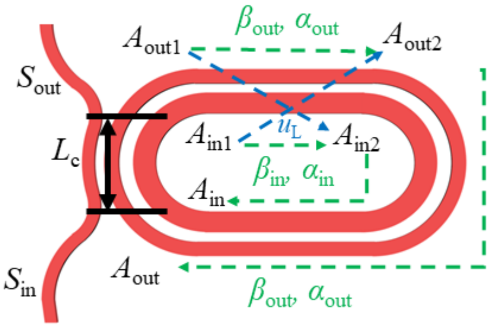

Fig. S5 | Schematic diagram of the multimode concentric resonator. The input pump is injected into the bus waveguide and couples to the outer racetrack, then to the inner racetrack.

Table S3 | Parameters of the multimode concentric resonator.

mode	Resonant wavelength (nm)	Propagation loss α (dB/cm)	Coupling ratio $\|\kappa\|^2$ (s^{-1})	Coupling condition
In_{TE0}	1329.5	0.2	N.A.	N.A.
Out_{TE0}	1329.502	0.4	7.4×10^8	Near critical

Compared to the multimode single resonator, the multimode concentric resonator in Fig. S5 displays a unique transmission property. Because the inner resonator does not directly interact with the bus waveguide. Considering the TE0 mode in the outer resonator (Out_{TE0}) and TE0 mode in the inner resonator (In_{TE0}) with parameters listed in Table S3, we solved the equations (2a-2c)[1] in the paper and obtained the transmission spectra of the multimode concentric resonator with different mutual coupling coefficients u_t as shown in Fig. 1e of the paper.

Table S4 | Calculated loaded Q factors of the multimode concentric resonator for different mutual coupling coefficients u_t corresponding to Fig. 1e in the paper.

u_t (s^{-1})	Loaded Q factor (Out_{TE0})	Loaded Q factor (In_{TE0})
0	9.63×10^5	N.A.
1×10^9	1.07×10^6	2.41×10^6
2×10^9	1.2×10^6	2.11×10^6
3×10^9	1.29×10^6	1.93×10^6
4×10^9	1.34×10^6	1.83×10^6

We calculated the loaded Q factors and summarized them in Table S4. We can see that the loaded Q factor of the Out_{TE0} slightly enhances as u_t increases. It can be understood that the Out_{TE0} with higher propagation loss couples to the In_{TE0} with lower propagation loss. The In_{TE0} shares the propagation loss of the Out_{TE0}. Thus, the loaded Q factor of the Out_{TE0} increases. In addition, also for the lower propagation loss of the inner resonator, when the mutual coupling happens, the In_{TE0} can present a higher loaded Q factor than that of the Out_{TE0} without mutual coupling. In other words, two resonances with higher loaded Q factors than that of Out_{TE0} without mutual coupling can appear in one FSR as shown in Table S4. We found that both the Out_{TE0} and In_{TE0} can keep loaded Q factors over 10^6 after the interaction. That is, even though the mode coupling from the higher-order mode can highly decrease the loaded Q factor at TE0 mode, as present in the multimode single resonator, the existence of the two high-Q resonances at TE0 mode in the multimode concentric resonator can keep at least one high-Q resonance at TE0 mode in each free spectral range (FSR).

S3. Broadband high-Q multimode concentric resonator

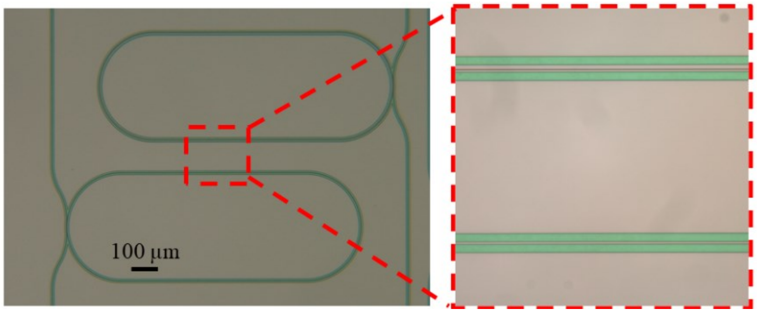

Fig. S6 | Microscope image of the multimode concentric resonator (up) and multimode single resonator (down). The zoomed-in image shows the clear views of two waveguides of the concentric resonator while one waveguide for the single resonator.

To further achieve broadband high Q factors in a multimode concentric resonator, a pulley directional coupler is useful. Because coupling coefficients with comparable values from 1200 nm to 1700 nm can be obtained by carefully engineering the pulley directional coupler. We then fabricated the multimode concentric resonators with the designed pulley directional coupler and compared them with the multimode single resonators without additional inner racetracks in Fig. S6. The two types of resonators were fabricated in the same chip to avoid fabrication errors for clear comparison.

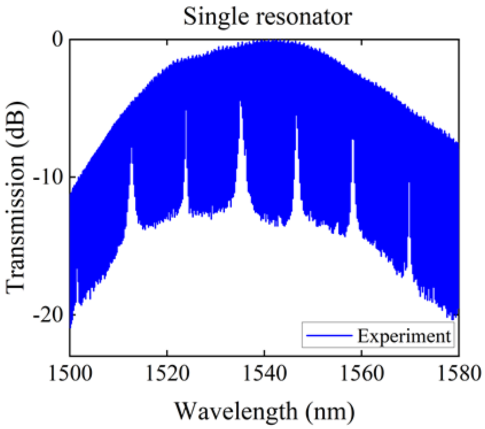

Fig. S7 | Experimental transmission spectrum of the multimode single resonator. The multimode single resonator shows the periodical mode coupling of the TE0 modes with the higher-order modes.

We first experimentally characterized the transmission spectra of the two resonators. Even employing the broadband pulley directional coupler, we found that the multimode single resonator still suffers from the effects of the higher-order modes periodically in Fig. S7. Because the higher-order modes can become nearly coincident with the fundamental modes periodically.

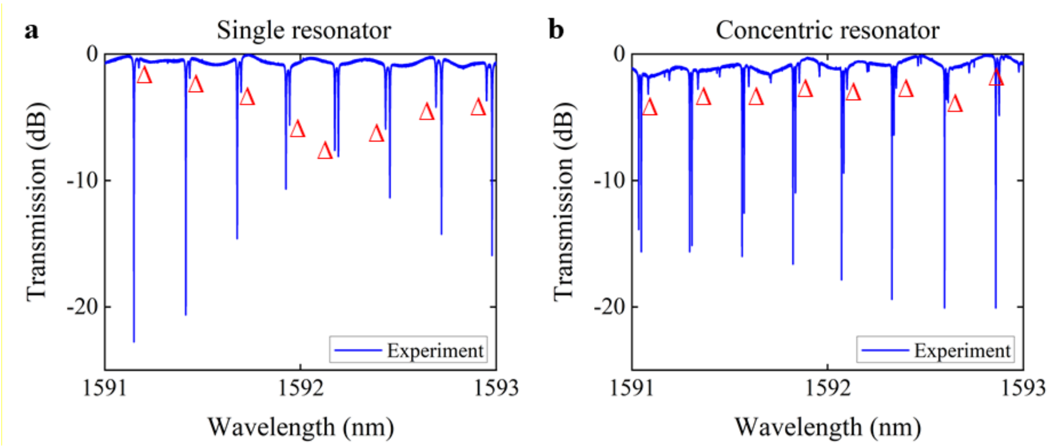

Fig. S8 | Experimental transmission spectra in the two resonators. a, In the multimode single resonator. **b,** In the multimode concentric resonator. The triangle marks represent the higher-order modes.

We magnify the affected resonances of the multimode single resonator at the mode coupling region in Fig. S8a. As the higher-order mode (triangle mark) gets close to the TE0 mode, more loss is coupled into the TE0 mode which results in a high decrease in the extinction ratio and the loaded Q factor. However, for the same wavelength range of the multimode concentric resonator in Fig. S8b, due to the split of two high-Q TE0 resonances at 1591 nm, even though the higher-order mode slightly catches the right TE0 resonance, the left TE0 resonance is barely affected. The principle of concentric resonators mitigating loaded Q factor reduction can be stated as follow. When a TE0 mode couples to a higher-order mode with a much larger propagation loss in a multimode single resonator, the loaded Q factor would be reduced seriously. However, in a multimode concentric resonator, when Out_{TE0} couples to In_{TE0} with slightly smaller propagation loss, both consequently split resonances present higher loaded Q factors comparable to that of the main resonant mode without mutual coupling. Therefore, by adding an inner resonator, we add an additional high-Q resonance within one FSR when independent resonant wavelengths of Out_{TE0} and In_{TE0} approach each other. If one mode encounters a higher-order mode and suffers from a serious reduction in loaded Q factor, there is still another mode propagating mainly in another resonator with displaying a high loaded Q factor.

In detail, when a higher-order mode slightly couples to a TE0 mode, they share the coupling loss and propagation loss of each other. However, the propagation loss of the higher-order mode can be up to 8 times larger than that of the TE0 mode. Therefore, in the mode coupling region, the effective propagation loss of the TE0 mode would increase. Conventionally, in a multimode single resonator, the higher-order mode is unwanted and its coupling from the bus waveguide should be suppressed. The small coupling ratio of the higher-order mode can lower the effective coupling ratio of the TE0 mode when they couple

to each other and share coupling ratios. Therefore, if the reduced coupling loss of the TE0 mode is smaller than its increased propagation loss, the total loss of the TE0 mode still increases, leading to the reduced loaded Q factor. TE0 mode always presents the reduced loaded Q factor in the mode coupling region for near-critical and under coupling cases. On the contrary, when the reduced coupling loss of the TE0 mode is larger than its increased propagation loss, the enhancement of the loaded Q factor occurs. We observed such a phenomenon in experiments only when TE0 mode is seriously over coupled with a low extinction ratio of only 6 dB. For nonlinear application, the cavity usually would not be so seriously over coupled where the loaded Q factor is quite small. Even though it can get a higher loaded Q factor from the mode coupling, most resonances are still with low Q factors. Therefore, we didn't consider it here. In our designs, the main resonant mode which is away from the mode coupling region presents slightly over coupling with a large extinction ratio of 15 dB under a low pump power from the laser. We classify the slightly over-coupling with extinction ratio over 10 dB into the near-critical coupling of which the loaded Q factors are still high at the order of 10^6.

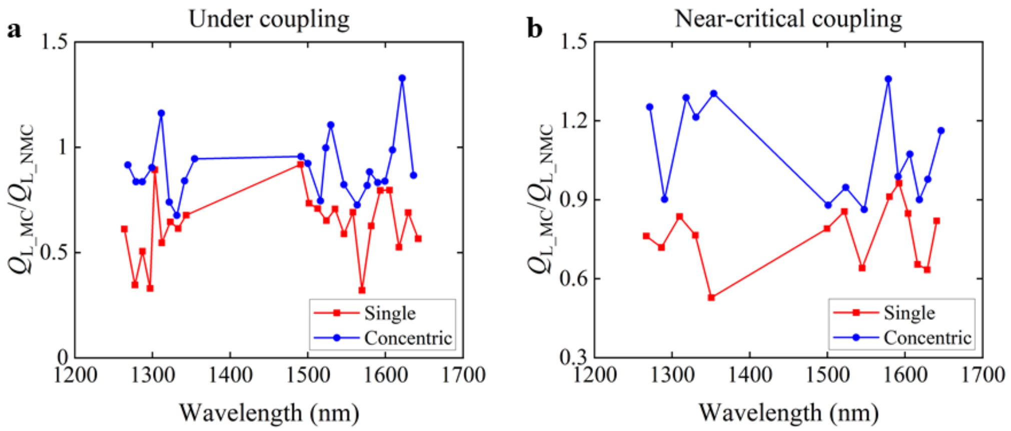

Fig. S9 | Loaded Q factors comparison between the multimode single and multimode concentric resonators. The ratio of loaded Q factor at mode coupling regions (Q_{L_MC}) over the nearby average loaded Q factors but slightly away from mode coupling regions (Q_{L_NMC}) from 1200 nm to 1700 nm in the multimode single and the multimode concentric resonators at **a,** under coupling and **b,** near-critical coupling conditions.

By varying the pulley directional couplers, we designed and fabricated two types of multimode single and multimode concentric resonators with under coupling and near-critical coupling in Fig. S9. We compared the ratio of the loaded Q factors at all the mode coupling regions over the nearby average loaded Q factors but slightly away from the mode coupling regions. At both under-coupling and near-critical coupling conditions, all the mode regions in the multimode concentric resonators exhibit higher loaded Q factor

ratios than those in the multimode single resonators, indicating smaller reductions of the loaded Q factors in the mode coupling regions.

As above mentioned, because of the mutual coupling from the inner resonator with lower propagation loss, two resonances with higher loaded Q factors than Out_{TE0} without mutual coupling can occur. If the higher-order mode affects one mode, there remains another mode presenting a high loaded Q factor. That is why Q_{L_MC}/Q_{L_NMC} can be larger than 1 in some mode coupling regions, as indicated in Fig. S9. We fabricated two pairs of the above devices in the same chip to consider the fabrication errors. All the devices can improve 97% loaded Q factors at all the mode coupling regions. The remaining 3% regions are the places where the mode coupling slightly affects the loaded Q factors. That is, the multimode concentric resonators can highly alleviate the reductions on loaded Q factors in the mode coupling regions. Besides, from Fig. S9, the near-critical coupling resonators can have less than 36% mode coupling regions compared to the under-coupling resonators. Generally, the multimode concentric resonators work well to mitigate the decrease of the loaded Q factors at the mode coupling regions and achieve the broadband high Q factors over a wide wavelength range.

To further validate our results experimentally, we fabricated two additional multimode concentric resonators. We kept the gaps between the bus waveguide and the outer racetracks as 400 nm. But we varied the gaps between the inner and outer racetracks as 500 nm and 300 nm in the two devices. Compared to the original device with a gap of 400 nm, the number of the mode coupling regions has increased by 45% and 19%. It may imply that the gap between the inner and outer racetracks is an important aspect to engineer for fewer mode coupling regions. Another two multimode concentric resonators with changing the width of the inner racetrack to 1.5 μm (the same width of the outer racetrack) and 3.25 μm were also fabricated in the same chip. The number of the mode coupling regions has increased by 15% and 6%. Very large reductions of the loaded Q factors in the multimode concentric resonator with a 1.5-μm-width inner racetrack were found in three of the mode coupling regions. A wide inner racetrack can reduce the loss, but it can also induce more higher-order modes. Thus, careful engineering of the widths of the inner racetrack is also critical. To reduce the higher-order modes in the multimode concentric resonator, other approaches, like single-mode bends, Euler bends, and Bezier bends are useful.

S4. Modes in the multimode concentric resonator

The simulated eigenstates of the supermodes in the concentric racetrack resonator are shown in Fig. S10. For the wavelength range from 1240 nm to 1680 nm, the TE2 mode in the multimode outer resonator is

strong at a short wavelength and gradually becomes less confined in the long wavelength. The inner resonator has a width of 3 μm which can support at least five modes as the below simulated mode profile. That is, the multimode concentric resonator is composed of multimode waveguides for both the outer and inner resonators.

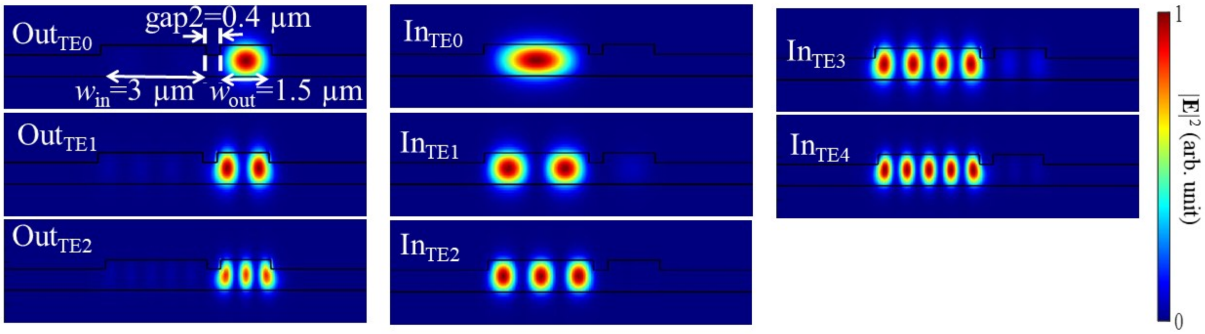

Fig. S10 | Simulated mode propagates mainly in the outer racetrack, and modes are excited in the inner racetrack. The mode profiles indicate that both the outer racetrack and the inner racetrack are multimode. The color bar indicates the intensity of the electric field |**E**|.

S5. Raman lasing characteristics

For resonators, maintaining a high Q factor is important in some nonlinear devices because the high Q can directly enable low-input power devices. The enhancement factor, denoting the largest build-up of light intensity in the cavity, is defined as the light intensity coupled into the ring over the light intensity in the bus waveguide[3]:

$$M = \frac{I_{\mathrm{p}}(0)}{I_{\mathrm{in}}} = \frac{1-\hat{t}^2}{(1-a\hat{t})^2} \tag{S10}$$

$\hat{t}$ is the transmission coefficient and a is the dimensionless loss coefficient. We calculated the ratios of the enhancement factors in the mode coupling region over the enhancement factors out of the mode coupling region in Fig. S11. Generally, the multimode concentric resonators exhibit smaller variation in enhancement factor than the multimode single resonators, since $M_{\mathrm{MC}}/M_{\mathrm{NMC}}$ is closer to one in Fig. S11 for the multimode concentric resonators. For the values of the enhancement factors, the multimode concentric resonators also manifest larger values compared to the multimode single resonators at the mode coupling regions.

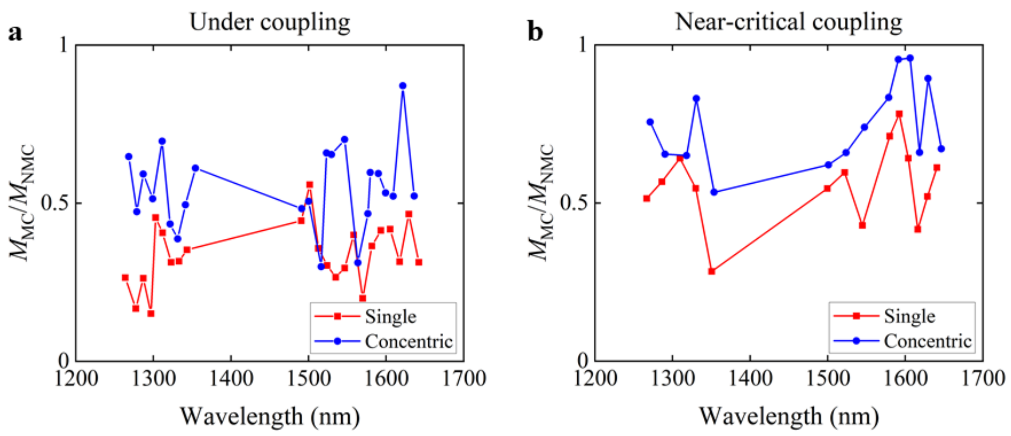

Fig. S11 | Enhancement factor comparison between the multimode single and multimode concentric resonators. The ratio of the enhancement factor at mode coupling regions (M_{MC}) over the nearby average enhancement factor but slightly away from mode coupling regions (M_{NMC}) from 1200 nm to 1700 nm in the multimode single and the multimode concentric resonators at **a,** under coupling and **b,** near-critical coupling conditions.

As an example of the benefit of using the multimode concentric resonator, we demonstrated its use in an integrated widely tunable Raman laser, where the enhancement factor directly affects the required input pump power to achieve Raman lasing threshold P_{th} as follows.

$$P_{th} = \frac{\alpha_s A_{eff}}{g_r M T} \tag{S11}$$

α_s is the linear loss coefficient for the Stokes, A_{eff} is the effective area, g_r is the Raman gain coefficient and T is the transmission factor (output power over input power).

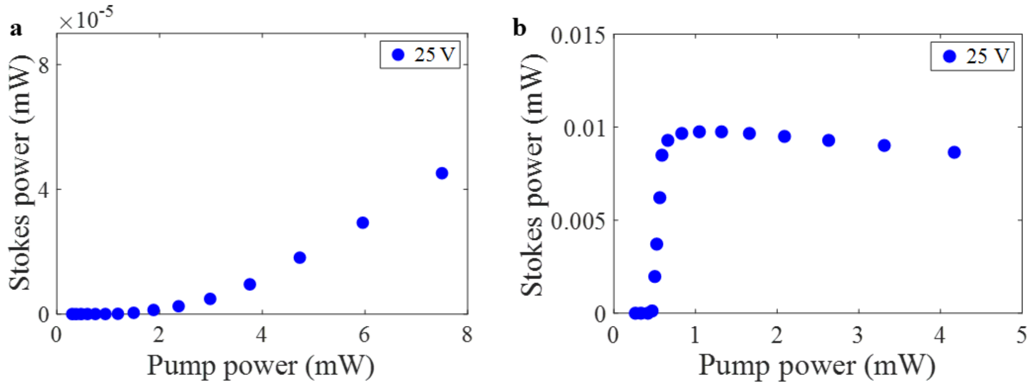

Fig. S12 | Raman lasing threshold power characteristics at the mode coupling regions. Stokes Raman laser output powers as a function of pump powers in **a**, multimode single resonator and **b**, multimode concentric resonator applied with 25 V reverse biases.

In the mode coupling region, we separately chose one resonance of the multimode single resonator and one resonance of the multimode concentric resonator to compare their Raman lasing threshold powers. They have corresponding enhancement factors as 50.8 and 15.3. Using equation S11, we can calculate the Raman lasing threshold power as 0.3 mW and 1.3 mW. Fig. S12 shows the Raman lasing threshold powers were measured as 0.5 mW and 1.5 mW, in consonance with the theoretical predictions. Besides, due to the highly decreased Q factor in the mode coupling region of the multimode single resonator, the Stokes output power is largely reduced by 500 times smaller at the similar pump powers. If using the highly affected resonance with the largely decreased Q factor in Fig. S4, the theoretical threshold power increases to 94 mW, which is 200 times larger than that in the multimode concentric resonator.

S6. Raman lasing experimental setup

For the Raman lasing measurement, the pump light was injected from a tunable laser. A polarization controller was used to adjust the light into the quasi-transverse-electric polarization. Then the light was coupled into the device via the edge couplers and lensed fibers. A 1:99 fiber coupler divided the light into a power meter to measure the output power and an optical spectrum analyzer to record the output spectrum.

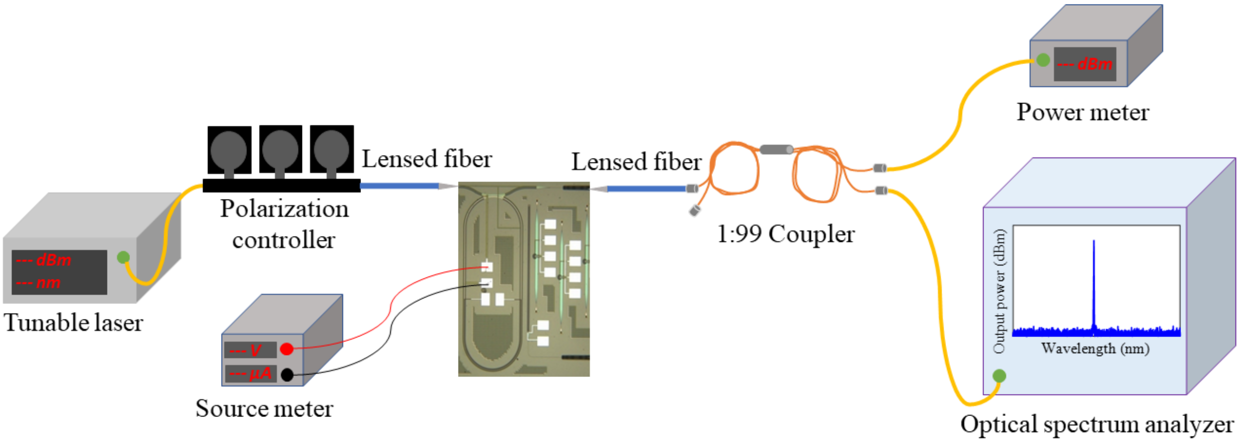

Fig. S13 | Experimental setup for Raman lasing measurement. The pump light from a tunable laser adjusted by a polarization controller is coupled into and out of the bus waveguide of the resonator via two lensed fibers. An optical spectrum analyzer records the output spectrum.

S7. Resonator characteristics

For an all-pass resonator, the loaded quality (Q_L) factor can be expressed as[4]:

$$Q_\mathrm{L} = \frac{\lambda}{\mathrm{FWHM}} = \frac{\pi n_\mathrm{g} L\sqrt{\hat{t}a}}{\lambda(1-\hat{t}a)}, \tag{S12}$$

where FWHM is the full width at half maximum of the resonance; n_g is the group velocity; a = exp($-\alpha L/2$) is the amplitude transmission and α is the power attenuation coefficient; L is the roundtrip length of the resonator; λ is the resonant wavelength; $\hat{t}$ is the self-coupling coefficient of the directional coupler. The intrinsic quality (Q_i) factor and coupling Q (Q_c) factor can be calculated by[5-7]:

$$Q_\mathrm{i} = \frac{2\pi n_\mathrm{g}}{\alpha\lambda} = \frac{2Q_\mathrm{L}}{1 \pm \sqrt{T_0}}, \tag{S13}$$

$$Q_\mathrm{c} = \omega \frac{n_\mathrm{g}}{c_0} \frac{2\pi R}{|\hat{\kappa}|^2} \tag{S14}$$

where ω is the resonant frequency; R is the radius of the resonator; T_0 is the normalized transmission at the resonance. Equation S13 takes – sign for over-coupled regime while + sign for under-coupled regime.

To satisfy the energy conservation, $\hat{t}^2 = 1 - \hat{\kappa}^2$, where $\hat{\kappa}$ is the cross-coupling coefficient of the directional coupler. To calculate $\hat{\kappa}$, a supermode approach is used[8]:

$$\hat{\kappa}(\lambda)^2 = \frac{\sin^2\left(u_\mathrm{L} \cdot L_\mathrm{dc} \cdot \sqrt{1 + (\frac{\Delta\beta}{2u_\mathrm{L}})^2}\right)}{1 + (\frac{\Delta\beta}{2u_\mathrm{L}})^2}, \tag{S18}$$

where $u_\mathrm{L} = \pi\Delta n/\lambda$ is coupling strength; L_dc is the length of the directional coupler; $\Delta\beta$ is the difference between the propagation constants of the bus waveguide and the outer racetrack. In the design, we only consider the eigenmode, $\mathrm{Out}_\mathrm{TE0}$, of the concentric racetracks, since $\mathrm{Out}_\mathrm{TE0}$ is directly coupled with the bus waveguide and hence can be seen in transmission even without mode coupling. If the power attenuation coefficient of $\mathrm{Out}_\mathrm{TE0}$ is assumed to be wavelength-independent, the required power coupling fraction from the bus waveguide to the concentric racetracks for loaded Q factor around 10^6 can be taken as a constant value, denoted as $\widehat{\kappa_\mathrm{c}}^2$. In other words, for a broadband resonator[9], $\mathrm{d}\widehat{\kappa_\mathrm{c}}^2/\mathrm{d}\lambda \approx 0$. If a symmetric directional coupler is applied, $\Delta\beta = 0$ and $\widehat{\kappa_\mathrm{c}}^2 = \sin^2(u_\mathrm{L} \cdot L_\mathrm{dc})$, meaning that $\widehat{\kappa_\mathrm{c}}^2$ depends totally on u_L. Because u_L is positively related to wavelength, i.e., the longer wavelength yields the less confined modes and more coupling. Therefore, it is difficult to get broadband critical coupling for the symmetric directional coupler. We adopt an asymmetric directional coupler here. Thus, a phase-mismatched condition is introduced to compensate for the change of u_L with wavelength.

S8. Comparison of silicon Raman lasers

In Table S5, we summarize the progress on integrated silicon Raman lasers from the pulsed Raman laser to continuous-wave Raman laser[10, 11]. Attempts on developing a low-threshold Raman laser and extending the lasing wavelength band from C-band to the mid-infrared region have also been included[3, 12]. A further approach to hybrid silicon with rare-earth can reduce the Raman lasing threshold power and extend the lasing wavelength to 1.9 μm, which is promising for mid-infrared applications[13].

Table S5 | Comparison between integrated silicon Raman lasers.

work	Platform	Input type	Lasing threshold (W)	Lasing slope efficiency	Bias (V)	Lasing wavelength band	Raman order
[10]	Silicon	Pulse	9	8.5%	0	C band	-
[11]	Silicon	cw	0.182	4.3%	25	C band	-
[3]	Silicon	cw	0.02	28%	25	C band	-
[12]	Silicon	cw	-	-	-	Mid-infrared	3
[13]	Hybrid rare-earth silicon	cw	0.016	4.2%	0	1.9 μm	-
This work	Silicon	cw	$(0.4 \pm 0.1) \times 10^{-3}$	$(8.5 \pm 1.5)\%$	25	C band	-

-: not reported in the paper.

S9. Photocurrent characteristics

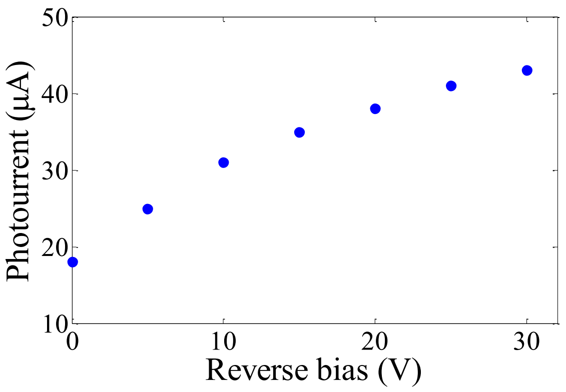

Fig. S14 | Photocurrent as a function of reverse bias voltage. The resonant wavelength is 1550 nm, and the pump power is 0.3 mW.

Choosing the pump power around the lasing threshold of 0.3 mW, we measure the photocurrents with reverse bias voltages from 0 V to 30 V in Fig. S14. The photocurrents increase rapidly from 0 V to 15 V and are prone to saturate after 25 V. Therefore, we adopt 25 V reverse bias voltages in the Raman lasing measurements.

S10. Development of low-loss silicon nano waveguide

The 450-nm SOI waveguides fabricated by a commercial multi-project wafer foundry have standard propagation losses of around 2 dB/cm. The wider multimode waveguides offer smaller propagation losses for the fundamental mode because of the reduced modal overlap with the sidewall roughness. Therefore, our fabricated multimode waveguide racetrack resonators can achieve loaded Q factors over 10^6. Apart from the multimode structure, recent progress on nano waveguides can achieve propagation loss as low as 0.4 dB/cm by optimizing the fabrication such as the etchless thermal oxidation[14, 15], high-resolution ArF immersion lithography[16], and post-etching roughness removal processes[17]. They are summarized in Table S6. We used the commercial foundry for fabrication, so we used the multimode waveguide to obtain low loss and further achieved a high-Q resonator.

Table S6 | Development of low-loss silicon nano waveguide.

Year	Width (nm)	Height (nm)	Slab (nm)	Wavelength (nm)	Loss (dB/cm)	Method
2011[14]	600	125	40	1600	0.35	Thermal oxidation with HSQ mask
2012[15]	300	500	\	1533	0.9	Thermal oxidation with LPCVD grown SiN mask
2016[16]	440 (320)	220	\	1550 (1310)	0.4 (1.28)	ArF immersion lithography
2020[17]	400	300	\	1550 (1300)	0.7 (1.1)	Si_3N_4 mask and H_2 thermal annealing

References

1. Zhang Z, Dainese M, Wosinski L, Qiu M. Resonance-splitting and enhanced notch depth in SOI ring resonators with mutual mode coupling. *Opt. Express* **16,** 4621-4630 (2008).
2. Okamoto K. *Fundamentals of optical waveguides*. Elsevier, (2021).
3. Rong H, Xu S, Kuo Y-H, Sih V, Cohen O, Raday O*, et al.* Low-threshold continuous-wave Raman silicon laser. *Nat. Photonics* **1,** 232 (2007).
4. Bogaerts W, De Heyn P, Van Vaerenbergh T, De Vos K, Kumar Selvaraja S, Claes T*, et al.* Silicon microring resonators. *Laser Photonics Rev.* **6,** 47-73 (2012).
5. Xuan Y, Liu Y, Varghese LT, Metcalf AJ, Xue X, Wang P-H*, et al.* High-Q silicon nitride microresonators exhibiting low-power frequency comb initiation. *Optica* **3,** 1171-1180 (2016).
6. Moille G, Li Q, Briles TC, Yu S-P, Drake T, Lu X*, et al.* Broadband resonator-waveguide coupling for efficient extraction of octave-spanning microcombs. *Opt. Lett.* **44,** 4737-4740 (2019).
7. Moille G, Perez EF, Stone JR, Rao A, Lu X, Rahman TS*, et al.* Ultra-broadband Kerr microcomb through soliton spectral translation. *Nat. Commun.* **12,** 1-9 (2021).
8. Chrostowski L, Hochberg M. *Silicon photonics design: from devices to systems*. Cambridge University Press, (2015).
9. Arlotti C, Gauthier-Lafaye O, Monmayrant A, Calvez S. Achromatic critically coupled racetrack resonators. *JOSA B* **34,** 2343-2351 (2017).
10. Boyraz O, Jalali B. Demonstration of a silicon Raman laser. *Opt. Express* **12,** 5269-5273 (2004).
11. Rong H, Jones R, Liu A, Cohen O, Hak D, Fang A*, et al.* A continuous-wave Raman silicon laser. *Nature* **433,** 725 (2005).
12. Jalali B, Raghunathan V, Shori R, Fathpour S, Dimitropoulos D, Stafsudd O. Prospects for silicon mid-IR Raman lasers. *IEEE J. Sel. Top. Quantum Electron.* **12,** 1618-1627 (2006).
13. Miarabbas Kiani K, Frankis HC, Naraine CM, Bonneville DB, Knights AP, Bradley JD. Lasing in a hybrid rare - earth silicon microdisk. *Laser Photonics Rev.* **16,** 2100348 (2022).
14. Nezhad MP, Bondarenko O, Khajavikhan M, Simic A, Fainman Y. Etch-free low loss silicon waveguides using hydrogen silsesquioxane oxidation masks. *Opt. Express* **19,** 18827-18832 (2011).
15. Griffith A, Cardenas J, Poitras CB, Lipson M. High quality factor and high confinement silicon resonators using etchless process. *Opt. Express* **20,** 21341-21345 (2012).
16. Horikawa T, Shimura D, Mogami T. Low-loss silicon wire waveguides for optical integrated circuits. *MRS Commun.* **6,** 9-15 (2016).
17. Wilmart Q, Brision S, Hartmann J-M, Myko A, Ribaud K, Petit-Etienne C*, et al.* A complete Si photonics platform embedding ultra-low loss waveguides for O-and C-band. *J. Lightwave Technol.* **39,** 532-538 (2020).
