# Supplementary material for: Broadband high-Q multimode silicon concentric racetrack resonators for widely tunable Raman lasers
Source: Nat Commun. 2022 Jun 20;13:3534. doi: 10.1038/s41467-022-31244-0 (PMC9209424; doi:10.1038/s41467-022-31244-0)
Supplement: Supplementary file 3 — Lasing Reporting Summary [file 41467_2022_31244_MOESM3_ESM.pdf]

Corresponding author(s): Hon Ki Tsang

Lasing Reporting Summary

Nature Research wishes to improve the reproducibility of the work that we publish. This form is intended for publication with all accepted papers reporting claims of lasing and provides structure for consistency and transparency in reporting. Some list items might not apply to an individual manuscript, but all fields must be completed for clarity.

For further information on Nature Research policies, including our data availability policy, see Authors & Referees.

Experimental design

Please check: are the following details reported in the manuscript?

1. Threshold

Plots of device output power versus pump power over a wide range of values indicating a clear threshold

☒ Yes
☐ No

The plots of output power versus pump power are clear shown in Fig. 3 and Fig. S12 (Supplementary Information). The related analyses are included in section "Broadband tunable Raman silicon laser".

2. Linewidth narrowing

Plots of spectral power density for the emission at pump powers below, around, and above the lasing threshold, indicating a clear linewidth narrowing at threshold

☒ Yes
☐ No

The lasing spectra are shown in Fig. 4c. The linewidth narrowing was confirmed by experiment measurement - The linewidth of the Raman laser was measured to be over two orders narrower than the resonator linewidth in two independent experimental measurements based on self-homodyne measurement with a delay interferometer and heterodyne linewidth measurement using a second tunable laser as described in section "Broadband tunable Raman silicon laser".

Resolution of the spectrometer used to make spectral measurements

☒ Yes
☐ No

Broad tuning was measured using two optical spectrum analyzers of YokogawaAQ6370D (600 – 1700 nm) with a resolution of 0.02 nm and YokogawaAQ6375B (1200 – 2400 nm) a resolution of 0.05 nm were used to record the lasing spectra up to 1841 nm. Narrow laser linewidth down <1 MHz frequency resolution was measured by hetrodyne laser linewidth measurement using RF spectrum analyzer.

3. Coherent emission

Measurements of the coherence and/or polarization of the emission

☒ Yes
☐ No

We indicated the polarization in section "Broadband tunable Raman silicon laser" as "Pumping with quasi-transverse-electric polarization at 1550 nm wavelength with reverse biases of 25 V and 10 V".

4. Beam spatial profile

Image and/or measurement of the spatial shape and profile of the emission, showing a well-defined beam above threshold

☐ Yes
☒ No

We used the commercial lasers as "O-band tunable laser Keysight 81606A (Keysight Technologies corporation) and 1450 nm to 1680 nm using a C-band tunable laser Keysight 81608A (Keysight Technologies corporation) and a L-band tunable laser TSL 510 (SANTEC corporation)" in section "Broadband tunable Raman silicon laser". These commercial lasers are well-demonstrated with narrow linewidths. Since the output is from a waveguide coupled to single mode fiber, the mode profile measurement is not needed.

5. Operating conditions

Description of the laser and pumping conditions
Continuous-wave, pulsed, temperature of operation

☒ Yes
☐ No

We mentioned "The experimental setup is detailed in Section 6 of the Supplementary Information" in section "Broadband tunable Raman silicon laser". And describe it in ”S6. Raman lasing experimental setup“, Supplementary Information

Threshold values provided as density values (e.g. W cm^{-2} or J cm^{-2}) taking into account the area of the device

☒ Yes
☐ No

We mentioned: "The threshold densities are 833 W/mm2 and 1111 W/mm2" in section "Broadband tunable Raman silicon laser".

6. Alternative explanations

Reasoning as to why alternative explanations have been ruled out as responsible for the emission characteristics
e.g. amplified spontaneous, directional scattering; modification of fluorescence spectrum by the cavity

☐ Yes
☒ No

The theory and our experiment in section "Broadband tunable Raman silicon laser"and lasing spectra in Fig. 4c clearly show that our Raman laser is optical pumped laser.

7. Theoretical analysis

Theoretical analysis that ensures that the experimental values measured are realistic and reasonable
e.g. laser threshold, linewidth, cavity gain-loss, efficiency

☒ Yes
☐ No

We mentioned “The experimental results agreed well with the theoretical predications in Section 5 of the Supplementary Information” in section "Broadband tunable Raman silicon laser". And analyze them in "S5. Raman lasing characteristics" of the Supplementary Information

8. Statistics

Number of devices fabricated and tested

☒ Yes
☐ No

We stated that the number of compared devices and more different devices as: "To further validate our results experimentally, we fabricated two additional multimode concentric resonators." and "Another two multimode concentric resonators with changing the width of..." in "S3. Broadband high-Q multimode concentric resonator" of the Supplementary Information .

Statistical analysis of the device performance and lifetime (time to failure)

☒ Yes
☐ No

We have tested several devices with different designs and analyzed them as: "To further validate our results experimentally, we fabricated two additional multimode concentric resonators..." in S3. Broadband high-Q multimode concentric resonator" of the Supplementary Information. Since the lifetime is not relevant, we didn't include it. But the lifetime of a silicon Raman laser is well-known, the readers can find the information in our cited papers.
